# Diffusion tensor imaging in acute-to-subacute traumatic brain injury: a longitudinal analysis

**DOI:** 10.1186/s12883-015-0525-8

**Published:** 2016-01-11

**Authors:** Brian L. Edlow, William A. Copen, Saef Izzy, Khamid Bakhadirov, Andre van der Kouwe, Mel B. Glenn, Steven M. Greenberg, David M. Greer, Ona Wu

**Affiliations:** 1 Department of Neurology, Massachusetts General Hospital, Harvard Medical School, Boston, MA USA; 2 Department of Radiology, Massachusetts General Hospital, Harvard Medical School, Boston, MA USA; 3 Athinoula A. Martinos Center for Biomedical Imaging, Massachusetts General Hospital, Charlestown, MA USA; 4 Department of Physical Medicine and Rehabilitation, Spaulding Rehabilitation Hospital, Harvard Medical School, Boston, MA USA; 5 Department of Neurology, Yale-New Haven Hospital, Yale School of Medicine, New Haven, CT USA

**Keywords:** Traumatic axonal injury (TAI), Fractional anisotropy (FA), Corpus callosum, Disability rating scale (DRS)

## Abstract

**Background:**

Diffusion tensor imaging (DTI) may have prognostic utility in patients with traumatic brain injury (TBI), but the optimal timing of DTI data acquisition is unknown because of dynamic changes in white matter water diffusion during the acute and subacute stages of TBI. We aimed to characterize the direction and magnitude of early longitudinal changes in white matter fractional anisotropy (FA) and to determine whether acute or subacute FA values correlate more reliably with functional outcomes after TBI.

**Methods:**

From a prospective TBI outcomes database, 11 patients who underwent acute (≤7 days) and subacute (8 days to rehabilitation discharge) DTI were retrospectively analyzed. Longitudinal changes in FA were measured in 11 white matter regions susceptible to traumatic axonal injury. Correlations were assessed between acute FA, subacute FA and the disability rating scale (DRS) score, which was ascertained at discharge from inpatient rehabilitation.

**Results:**

FA declined from the acute-to-subacute period in the genu of the corpus callosum (0.70 ± 0.02 vs. 0.55 ± 0.11, *p* < 0.05) and inferior longitudinal fasciculus (0.54+/−0.07 vs. 0.49+/−0.07, *p* < 0.01). Acute correlations between FA and DRS score were variable: higher FA in the body (*R* = −0.78, *p* = 0.02) and splenium (*R* = −0.83, *p* = 0.003) of the corpus callosum was associated with better outcomes (i.e. lower DRS scores), whereas higher FA in the genu of the corpus callosum (*R* = 0.83, *p* = 0.02) corresponded with worse outcomes (i.e. higher DRS scores). In contrast, in the subacute period higher FA in the splenium correlated with better outcomes (*R* = −0.63, *p* < 0.05) and no inverse correlations were observed.

**Conclusions:**

White matter FA declined during the acute-to-subacute stages of TBI. Variability in acute FA correlations with outcome suggests that the optimal timing of DTI for TBI prognostication may be in the subacute period.

## Background

Traumatic axonal injury (TAI) is the most common [1], devastating [2], and difficult to detect [3] lesion in patients with traumatic brain injury (TBI). Computed tomography (CT) and conventional magnetic resonance imaging (MRI) have limited sensitivity for detecting the white matter shearing injury that is caused by rotational acceleration forces in civilian TAI [4] and blast forces in military TAI [5]. Diffusion tensor MRI (DTI) may improve detection of TAI by identifying disruptions in the axon bundles that are the neuroanatomic substrate of TAI [6, 7]. DTI measurements of reduced white matter fractional anisotropy (FA) have been correlated with histopathological evidence of TAI in experimental animal models of TBI [8, 9], as well as neurological deficits in human patients with mild, moderate, and severe TBI [10–13]. Furthermore, DTI measurements of FA have shown promise in improving the accuracy of prognostication for patients with TBI [14–16].

Despite increasing use of FA as a diagnostic and prognostic biomarker of brain injury severity in patients with TBI, the optimal timing of DTI data acquisition for TBI outcome prediction has yet to be determined. In the subacute [11, 16–18] and chronic [10, 11, 19–21] stages of TBI, low white matter FA consistently correlates with poor outcomes, as determined by the Glasgow Outcome Scale score [11, 17, 18, 20] or neurocognitive tests [10, 19, 21]. However, in the acute stage of TBI, white matter FA may increase or decrease due to the heterogeneous effects of intracellular and extracellular edema on local water diffusion [22, 23]. Accordingly, there are conflicting data on the relationship between acute FA and functional outcomes: acute increases in FA [24, 25] and acute decreases in FA [12, 13] may both be associated with poor outcome. Clarification of the time course of FA changes in the acute-to-subacute stages of TBI is therefore critical for understanding the pathophysiology of TAI, as well as for determining the optimal timing of DTI for TBI outcome prediction. The purpose of this study is to investigate longitudinal FA changes in a population of patients with complicated mild (Glasgow Coma Scale [GCS] score 13–15 with abnormal CT), moderate (GCS 9–12), and severe (GCS 3–8) TBI who underwent DTI scans during both the acute and subacute stages of injury. We hypothesized that white matter FA declines between the acute and subacute periods and that subacute FA correlates with functional outcome more consistently than does acute FA.

## Methods

### Participants

Three-hundred fifty subjects were retrospectively identified from a single center’s prospective contribution to the TBI Model Systems National Database (TBIMS ND) from 1999 to 2007. The TBIMS ND is a multicenter, longitudinal TBI study funded by the U.S. National Institute on Disability and Rehabilitation Research. TBIMS ND enrollees were 16 years or older, received TBI care in a TBIMS-affiliated hospital within 24 h of injury, and were transferred directly from acute care to an affiliated inpatient rehabilitation hospital (RH). Patients enrolled in the TBIMS ND study underwent a Disability Rating Scale (DRS) assessment that was performed by a certified examiner on the day of RH discharge. The DRS score incorporates assessments of arousal and awareness, ability to perform self-care, level of physical dependence, and psychosocial capacity for work or school [26]. Total DRS score ranges from 0 (no disability) to 29 (vegetative state). All study procedures were approved by the Spaulding Rehabilitation Hospital Institutional Review Board. Written informed consent was obtained from all study participants or next-of-kin surrogate decision-makers if study participants were deemed unable to provide consent. A separate research protocol was approved by the Massachusetts General Hospital Institutional Review Board for a retrospective review of clinical records and DTI scans of patients enrolled in the Spaulding Rehabilitation Hospital database who were treated for acute TBI at Massachusetts General Hospital, where DTI was routinely performed.

All local TBIMS database enrollees who underwent an acute (day 1–7) and a subacute (day 8 to RH discharge) DTI scan were eligible for inclusion in this study. Our definition of the acute and subacute time periods was based on prior TBI imaging studies [13, 25, 27]. Of the 350 subjects in our local TBIMS database, 146 were treated for acute TBI at our hospital from 2003 to 2007, which is the period during which DTI was routinely performed with all clinical MRI scans. Thirteen of these 146 patients underwent both an acute and a subacute DTI scan. All DTI scans were evaluated by a neuroradiologist for data quality, and clinical records were reviewed to identify neurologic events that could confound DTI measurements of FA. Two patients were excluded because of infarcts diagnosed in close temporal proximity to their TBIs.

All MRIs were performed at the discretion of the treating clinicians. The 11 acute MRI scans were ordered to diagnose intracranial pathology relating to the TBI. The 11 subacute MRI scans were ordered for prognostication (*n* = 4), suspected intracranial or subgaleal infection (*n* = 5), change in mental status (*n* = 1), and left arm weakness (*n* = 1). No new parenchymal lesions were identified on the subacute scans. One of the 11 patients did not have a DRS score assessed at the time of RH discharge.

### Image acquisition and processing

The 22 patient MRI scans analyzed in this study were obtained on multiple 1.5 Tesla scanners (General Electric Medical Systems, Waukesha, WI), which were upgraded several times at our institution over the 4-year study period. The DTI sequence was either a single-shot, spin-echo echo-planar imaging (SE-EPI) sequence (*n* = 10) or a twice refocused SE-EPI sequence [28] (*n* = 12). Other acquisition parameters are shown in Table 1. For nine patients (patients 1–9), 6 diffusion-encoding directions were used for both the acute and subacute DTI scans. For two patients (patients 10 and 11), the acute DTI scan utilized 6 diffusion-encoding directions repeated three times, whereas the subacute DTI scan utilized 25 directions. All DTI data were eddy-current corrected to minimize motion artifact and post-processed using techniques previously described [29].

**Table 1:** DTI data acquisition parameters (*n* = 11 TBI patients, *n* = 22 DTI scans)

ID	Post-TBI scan days	Field strength	FOV (mm)	*b* value (s/mm^2^)	# Directions	# *b*0	NEX	TR (msec)	TE (msec)	In-plane resolution (mm)	Slice thickness (mm)	Inter-slice gap (mm)	Matrix
1	5	1.5 T	220	1000	6	1	3	7500	99.3	1.72 × 1.72	5	1	128 × 128
	14	1.5 T	220	1000	6	1	3	7500	99.2	1.72 × 1.72	6	1	128 × 128
2	2	1.5 T	220	1000	6	1	3	7500	99.2	1.72 × 1.72	5.5	1	128 × 128
	13	1.5 T	220	1000	6	1	3	7500	72.9	1.72 × 1.72	5	1	128 × 128
3	1	1.5 T	220	1000	6	1	3	7500	99.3	1.72 × 1.72	5	1	128 × 128
	29	1.5 T	220	1000	6	1	3	7500	72.9	1.72 × 1.72	5	1	128 × 128
4	1	1.5 T	220	1000	6	1	3	7500	99.3	1.72 × 1.72	5	1	128 × 128
	19	1.5 T	220	1000	6	1	3	7500	99.3	1.72 × 1.72	5	1	128 × 128
5	2	1.5 T	220	1000	6	1	5	5000	88.9	0.86 × 0.86	5	1	128 × 128^a^
	97	1.5 T	220	1000	6	1	5	5000	95.7	0.86 × 0.86	5	1	128 × 128^a^
6	2	1.5 T	220	1000	6	1	3	7500	99.2	1.72 × 1.72	5	1	128 × 128
	11	1.5 T	220	1000	6	1	5	5000	99.4	0.86 × 0.86	5	1	128 × 128^a^
7	5	1.5 T	220	1000	6	1	5	5000	88.9	0.86 × 0.86	5	1	128 × 128^a^
	17	1.5 T	220	1000	6	1	5	5000	88.9	0.86 × 0.86	5	1	128 × 128^a^
8	2	1.5 T	220	1000	6	1	5	5000	91.9	0.86 × 0.86	5	1	128 × 128^a^
	32	1.5 T	260	1000	6	1	5	7000	91.3	1.02 × 1.02	5	1	128 × 128^a^
9	7	1.5 T	220	1000	6	1	5	5000	96.9	0.86 × 0.86	5	1	128 × 128^a^
	15	1.5 T	220	1000	6	1	5	5000	91.9	0.86 × 0.86	5	1	128 × 128^a^
10	2	1.5 T	220	1000	6	1	5	5000	91.9	0.86 × 0.86	5	1	128 × 128^a^
	19	1.5 T	220	1000	25	3	1	5000	78.8	0.86 × 0.86	5	1	128 × 128^a^
11	5	1.5 T	220	1000	6	1	3	7500	99.3	1.72 × 1.72	5	1	128 × 128
	38	1.5 T	220	1000	25	3	1	5000	85.3	0.86 × 0.86	5	1	128 × 128^a^

Abbreviations: *b0 b* = 0 s/mm^2^, *FOV* field of view, *NEX* number of excitations (i.e. number of averages), *T* Tesla, *TBI* traumatic brain injury, *TE* echo time, *TR* repetition time, # number of. ^a^Acquisition matrix was 128 x 128, zero-filled to 256 × 256

### Region of interest (ROI) analyses

ROIs were manually traced on the *b*0 images by an investigator blinded to the clinical data using Analyze 10.0 image display software (Mayo Clinic Biomedical Imaging Resource, Rochester, Minnesota, USA). Confirmation of ROI neuroanatomic localization was performed by a board-certified neuroradiologist, after which the mean and standard deviation values for FA and the apparent diffusion coefficient (ADC) were measured in each ROI. All ROIs were elliptical, and ROI dimensions were kept constant for bilateral ROIs and for each ROI across subjects.

**Fig. 1:**
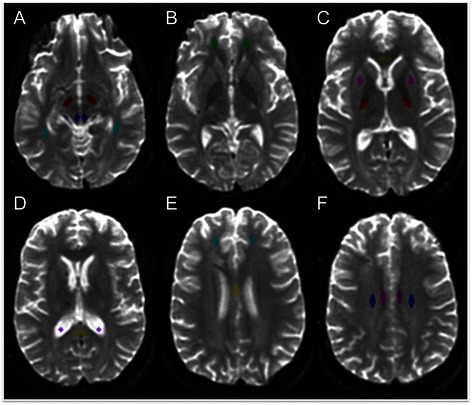
Neuroanatomic localization of regions of interest on *b*0 images. **a** Dorsolateral midbrain (*blue*), cerebral peduncle (*maroon*), and inferior longitudinal fasciculus (*turquoise*); **b** uncinate fasciculus (*green*); **c** genu of corpus callosum (*yellow*), posterior limb of the internal capsule (*red*), and putamen (*pink*); **d** splenium of the corpus callosum (*yellow*) and cerebrospinal fluid (*purple*); **e** anterior corona radiata (*turquoise*) and body of the corpus callosum (*yellow*); **f** cingulum bundle (pink) and centrum semiovale (*blue*)

Eleven white matter ROIs were selected for analysis of FA and ADC (Fig. 1): genu of the corpus callosum (CC), body of the CC, splenium of the CC, posterior limb of the internal capsule, uncinate fasciculus, anterior corona radiata, centrum semiovale, cingulum bundle, inferior longitudinal fasciculus, cerebral peduncle, and dorsolateral midbrain. All ROIs were chosen on the basis of prior studies demonstrating an association between FA and functional or cognitive outcomes in TBI [10–13, 17–19, 24], except for the dorsolateral midbrain ROI, which was chosen on the basis of data demonstrating diffusivity changes in the brainstem tegmentum in patients with traumatic disorders of consciousness [30, 31]. The putamen and CSF were included as internal control ROIs, since FA in these regions should not be affected by TAI [11, 13].

### Statistical analyses

Statistical analyses were performed using R version 2.11.1. Since TAI may cause asymmetric white matter injury [32, 33], FA and ADC values in bilateral ROIs were compared using a two-tailed Wilcoxon signed-rank test. If a contusion or susceptibility artifact from intracranial hardware overlapped with an ROI, the ROI was excluded from analysis. When the excluded ROI was a midline structure (i.e. genu, body or splenium of the CC), the ROI was excluded from all longitudinal and correlation analyses for that subject. When one side (left or right) of a bilateral ROI was excluded at a single time point, only the diffusion measurements from the contralateral side were used in the longitudinal and outcome correlation analyses. No laterality effect on FA or ADC was identified (*p* > 0.05 for all bilateral ROIs); thus, left- and right-sided values were averaged.

Differences in acute and subacute FA and ADC were tested using a two-tailed paired Wilcoxon signed-rank test. To determine if variability in the timing of DTI data acquisition modulated any statistically significant longitudinal FA changes, we tested for correlations between the number of days between the acute and subacute DTI scans and the change in FA using a two-tailed Spearman correlation test. Correlations between acute FA, subacute FA, and DRS were assessed for each white matter ROI using a two-tailed Spearman correlation test. Intrarater and interrater reliability of FA measurements were tested using the intraclass correlation coefficient for the ROIs that demonstrated significant longitudinal changes or correlations with outcome in the patient cohort. Raters were treated as a random factor. For the intrarater analysis, ROIs were placed by the same rater at least 4 weeks apart. For the interrater analysis, we calculated intraclass correlation coefficients for all 22 DTI scans, the 11 acute DTI scans, and the 11 subacute DTI scans. The latter two analyses were performed to determine whether the timing of DTI data acquisition affected the reproducibility of the FA measurements. We also used the two-tailed Spearman’s correlation coefficient to test whether correlations between regional FA and DRS persisted when FA was measured by ROIs drawn by the second rater.

### Healthy control DTI data

Since DTI data in our retrospective patient cohort were obtained with variable hardware (i.e. different MRI scanners) and software (i.e. different DTI sequence), we considered the possibility that this variability may have confounded our longitudinal measurements of changes in FA [34]. Some investigators have proposed acquiring control DTI data using the same scanner and sequence used for the patient cohort and then normalizing patient FA to control FA for each ROI [14], but no such control dataset was available for our retrospective patient cohort. We therefore prospectively acquired DTI data in a healthy control subject to measure the repeatability and variability of regional FA measurements. The control subject was a healthy man who underwent six DTI scans on a 1.5 Tesla Siemens MRI scanner and three DTI scans on three different 3 Tesla Siemens MRI scanners (Siemens Medical Solutions, Erlangen, Germany) over a period of 6 years (age 30 to 36). The healthy volunteer provided written informed consent in accordance with a research protocol approved by the Massachusetts General Hospital Institutional Review Board.

The six DTI scans on the single 1.5 Tesla Siemens MRI scanner were performed during three separate scanning sessions over a 2 h period (the subject was removed from the scanner inbetween scans). The three DTI scans on the 3 Tesla MRI scanners were performed on different days. The parameters of interest in all nine control DTI scans were varied to either match or fall within the range of the number of diffusion-encoding directions and spatial resolution (voxel size) of the DTI parameters used in the patient cohort (see Table 2). All other parameters (e.g. repetition time, echo time, etc.) were matched as closely as possible to those of the patient DTI scans. To reduce the number of statistical comparisons, we performed FA analyses in the control DTI scans only in the ROIs that demonstrated significant longitudinal changes or correlations with outcome in the patient cohort. We calculated the coefficient of variation (CV = standard deviation/mean) and reproducibility coefficient (RDC = 2.77*standard deviation), both measures of precision for imaging biomarkers [35]. We then compared FA variability in the control subject with longitudinal FA changes in the TBI patients to determine if the mean difference of the patients’ longitudinal FA changes exceeded the expected FA RDC (the 95 % precision limit) for each region.

**Table 2:** Healthy control DTI data acquisition parameters (*n* = 1 subject, *n* = 9 DTI scans)

Scanning session	DTI scan #	Field strength	FOV (mm)	*b* value (s/mm^2^)	# Directions	# *b*0	NEX	TR (msec)	TE (msec)	In-plane resolution (mm)	Slice thickness (mm)	Inter-slice gap (mm)	Matrix
1	1	3 T	256	700	12	2	5	8000	84	2.0 × 2.0	5	1.0	128 × 128
2	2	3 T	220	1000	25	3	1	4800	91	1.4 × 1.4	5	1.0	160 × 160
3	3	3 T	220	1000	25	3	3	5300	108	1.4 × 1.4	5	1.0	160 × 160
4	4	1.5 T	220	1000	6	1	3	5000	88	1.72 × 1.72	5.0	1.0	128 × 128
5	5	1.5 T	220	1000	6	1	3	5000	88	1.72 × 1.72	5.0	1.0	128 × 128
	6	1.5 T	220	1000	6	1	3	5000	88	1.72 × 1.72	5.0	1.0	128 × 128
6	7	1.5 T	220	1000	6	1	3	5000	88	1.72 × 1.72	5.0	1.0	128 × 128
	8	1.5 T	220	1000	6	1	3	9400	88	2.0 × 2.0	2.0	0	110 × 110
	9	1.5 T	220	1000	25	3	1	5000	88	1.72 × 1.72	5.0	1.0	128 × 128

The control subject’s first three DTI scans were performed on three different 3 Telsa MRI scanners on different days, whereas the next six DTI scans were performed on a 1.5 Tesla MRI scanner on a single day during three separate scanning sessions over a 2-hour period. Abbreviations: *b*0 *b* = 0 s/mm^2^, *FOV* field of view, *NEX* number of excitations (i.e. number of averages), *T* Tesla, *TE* echo time, *TR* repetition time, *#* number of

## Results

### Participant demographics and clinical characteristics

Demographic and clinical data are summarized in Table 3. The patient cohort was comprised of eight men and three women. Mean (SD) age was 36.2 (19.7) years, with a range of 16 to 77 years. All patients had at least one contusion on the admission head CT scan, and ten of the 11 patients underwent one or more neurosurgical intervention: intracranial bolt placement, hemicraniectomy, or external ventricular drain placement. One patient had a unilateral non-reactive pupil on admission (patient 7). None had a spinal cord injury. The acute DTI scan was performed on [mean +/− SD] day 3.1 +/− 1.9, and the subacute DTI scan was performed on day 27.6 +/− 23.4. Of note, FA and ADC could not be measured from the following ROIs in at least one DTI scan, because of overlapping contusion or susceptibility artifact from intracranial hardware: left CP (*n* = 1), right CP (*n* = 1), left UF (*n* = 7), and right UF (*n* = 8), left PL IC (*n* = 1), right PL IC (*n* = 1), left ACR (*n* = 5), right ACR (*n* = 11), genu of the CC (*n* = 5), body of the CC (*n* = 3), right CB (*n* = 2), left CS (*n* = 2), right CS (*n* = 1), left putamen (*n* = 1), and right putamen (*n* = 6).

**Table 3:** Demographic and clinical information

Patient ID	GCS	Mechanism	CT Classification	IMPACT Score (favorable)	Neurosurgery	Acute DTI day	Subacute DTI day	DRS score	RH D/C day
1	10	MVA	Diffuse injury IV	63 %	bolt	5	14	2.5	63
2	6	MVA	Diffuse injury II	62 %	bolt	2	13	11	241
3	13	fall	Evacuated mass lesion	68 %	bolt, crani	1	29	4	53
4	7	MVA	Diffuse injury II	83 %	none	1	19	3.5	45
5	6	MVA	Diffuse injury III	62 %	bolt	2	97	4.5	122
6	6	MVA	Diffuse injury II	88 %	EVD	2	11	4.5	102
7	5	MVA	Evacuated mass lesion	45 %	crani, bolt, EVD	5	17	5.5	363
8	14	Fall	Evacuated mass lesion	61 %	EVD	2	32	--	66
9	7	MVA	Diffuse injury III	81 %	EVD	7	15	1	31
10	3	MVA	Evacuated mass lesion	67 %	crani, bolt	2	19	23	178
11	15	fall	Diffuse injury II	44 %	crani	5	38	8.5	108
Summary	7 (3–15)	8 MVA	4 diffuse II	66.0 ± 13.5 %	6 bolt	3.1 +/− 1.9	27.6 +/− 23.4	4.5 (1–23)	124.7 +/− 96.0
			2 diffuse III		4 EVD				
		3 Fall	1 diffuse IV		4 crani				
			4 evacuated mass		1 none				

Summary data in the bottom row are provided as mean +/− standard deviation except for GCS and DRS scores, which are reported as median (range). Abbreviations: *crani* hemicraniectomy, *DTI* diffusion tensor imaging, *EVD* external ventricular drain, *DRS* disability rating scale, *GCS* admission Glasgow Coma Scale score, *MVA* motor vehicle accident, *RH* rehabilitation hospital, *TBI* traumatic brain injury. The IMPACT score is reported for the complete IMPACT model (core + CT + lab data elements), based upon clinical, imaging, and laboratory data recorded at the time of admission. Scores were calculated using the online IMPACT prognostic calculator (http://www.tbi-impact.org/?p=impact/calc). Of note, we report the IMPACT model’s predicted probability of a favorable outcome at 6 months. Subject 8 did not have DRS data recorded (−−) at the time of RH discharge

### Longitudinal DTI results

**Fig. 2:**
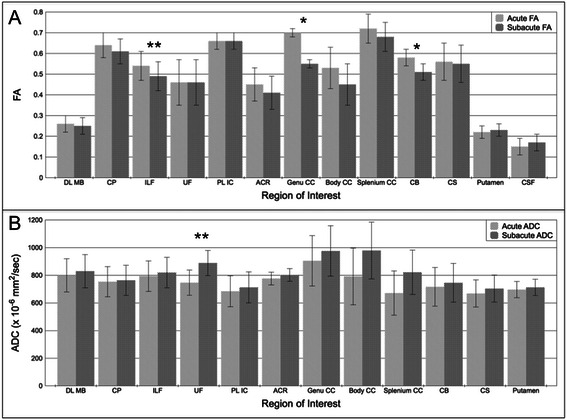
Longitudinal changes in FA and ADC. **a** Acute and subacute FA measurements, and **b** acute and subacute ADC measurements for each ROI. All data are displayed as mean +/− SD. Acute time period is defined as post-trauma days 1–7. Subacute period is defined as post-trauma day 8 until discharge from rehabilitation hospital. Acute and subacute diffusion parameters were compared using a two-tailed paired Wilcoxon signed-rank test. *p < 0.05; **p < 0.01. Of note, the longitudinal FA change in CB was within the 95 % precision limit for the healthy control’s FA measurements in this region. The acute CSF ADC value (2729 +/− 461 x 10^−6^ mm^2^/s) and the subacute CSF ADC value (3049 +/− 231 x 10^−6^ mm^2^/s) are not shown in panel **b** because of the difference in scale between CSF ADC values and all other ADC values. Abbreviations: ACR, anterior corona radiata; CB, cingulum bundle; CP, cerebral peduncle; CS, centrum semiovale; CSF, cerebrospinal fluid; DL MB, dorsolateral midbrain; ILF, inferior longitudinal fasciculus; PL IC, posterior limb of internal capsule; UF, uncinate fasciculus

**Fig. 3:**
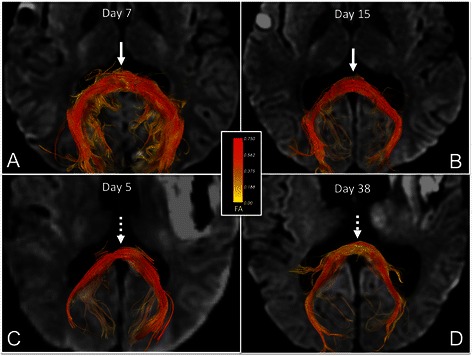
Longitudinal changes in fiber tract FA in the splenium of the corpus callosum. Superior view of acute (**a**) and subacute (**b**) fiber tracts of the splenium of the corpus callosum (CC) in a patient with good outcome (DRS = 1). Fiber tracts are color-coded according to mean FA (*center inset*) so that longitudinal splenium FA changes in each patient can be observed. Superior view of acute (**c**) and subacute (**d**) fiber tracts of the splenium of the CC in a patient with poor outcome (DRS = 8.5). In the patient with good outcome, splenium FA values are high in both the acute and subacute periods, as indicated by the red tract colors (*solid arrows*). In the subject with poor outcome, splenium FA values decline from the acute to the subacute period, as indicated by the red tracts in the acute period as compared to yellow tracts in the subacute period (*dotted arrows*). All tracts were reconstructed using Diffusion Toolkit version 0.6.2 and virtually dissected by manually tracing a splenium ROI in TrackVis version 5.2 (Wang & Wedeen, Athinoula A. Martinos Center for Biomedical Imaging, www.trackvis.org). Fiber tracts from the fornix and optic radiations were excluded to isolate the splenium fiber tracts. Tracts are superimposed on axial diffusion-weighted images at the level of the inferior aspect of the splenium of the CC

FA declined from the acute to the subacute period in the genu of the CC (acute FA [mean+/−SD] = 0.70+/−0.02, subacute FA = 0.55+/−0.11; *p* = 0.02), inferior longitudinal fasciculus (acute FA = 0.54+/−0.07, subacute FA = 0.49+/−0.07; *p* < 0.01) and cingulum bundle (acute FA = 0.58+/−0.04, subacute FA = 0.51+/−0.09; *p* = 0.02). The number of days between the acute and subacute DTI scans was not associated with the change in FA for any of these ROIs (*p* = 0.42 to 0.99). Of note, longitudinal FA changes in the genu of the corpus callosum and the inferior longitudinal fasciculus remained statistically significant when the two patients with different acute and subacute diffusion-encoding directions were excluded from the analysis (patients 10 and 11; *p* = 0.03 for both tests). However, the longitudinal FA change in the cingulum bundle was no longer statistically significant when these two subjects were excluded (*p* = 0.10). When the longitudinal FA change within the cingulum bundle was also reanalyzed without the two subjects for whom unilateral ROIs were excluded due to contusions or susceptibility artifacts, the acute-to-subacute FA change was no longer statistically significant: acute FA = 0.57+/−0.04, subacute FA = 0.51+/−0.09, *p* = 0.07. ADC increased from the acute to the subacute period in the uncinate fasciculus (acute ADC = 747+/−91 × 10^−6^ mm^2^/s, subacute ADC = 889+/−64 × 10^−6^ mm^2^/s; *p* = 0.008). Longitudinal FA and ADC data for all ROIs are provided in Fig. 2. Figure 3 shows the results of a post-hoc DTI tractography analysis that demonstrates the difference between acute-to-subacute splenium FA changes in a patient with good outcome (DRS = 1) and in a patient with poor outcome (DRS = 8.5).

### Correlations between DTI and functional outcome

In the acute period, decreasing FA in the genu of the CC correlated with better outcome (lower score) on the DRS (*R* = 0.83, *p* = 0.02), whereas increasing FA in the body of the CC (*R* = −0.78, *p* = 0.02) and splenium of the CC (*R* = −0.83, *p* = 0.003) correlated with better outcome. In the subacute period, increasing FA in the splenium of the CC correlated with better outcome on the DRS (*R* = −0.63, *p* = 0.049).

### Intrarater and interrater reliability analyses

In the intrarater reliability analysis for all 22 DTI scans, the intraclass correlation coefficients were: genu of the CC = 0.96, body of the CC = 0.96, splenium of the CC = 0.97, cingulum bundle = 0.93, and inferior longitudinal fasciculus = 0.94 (*p* < 0.001 for all analyses). In the interrater reliability analysis, the intraclass correlation coefficients were: genu of the CC [all = 0.93 (*p* = 0.001); acute = 0.53 (*p* = 0.07); subacute = 0.94 (*p* < 0.001)], body of the CC [all = 0.72 (*p* < 0.001); acute = 0.84 (*p* < 0.001); subacute = 0.60 (*p* = 0.03)], splenium of the CC [all = 0.93 (*p* < 0.001); acute = 0.92 (*p* = 0.002); subacute = 0.93 (*p* < 0.001)], cingulum bundle [all = 0.96 (*p* < 0.001); acute = 0.92 (*p* < 0.001); subacute = 0.95 (*p* < 0.001)], and inferior longitudinal fasciculus [all = 0.88 (*p* < 0.001); acute = 0.85 (*p* < 0.001); subacute = 0.88 (*p* < 0.001)]. When correlations between genu, body, and splenium FA and DRS score were retested using FA measurements generated by the second rater, we found similar correlations between DRS score and acute splenium FA (*R* = −0.63, *p* < 0.05), as well as between DRS score and subacute splenium FA (*R* = −0.67, *p* = 0.03). However, correlations between DRS score and acute FA in the body and genu of the CC were no longer significant (*p* > 0.05).

### Comparison of patient and control DTI data

For the control subject’s regional FA measurements and their reproducibility, see Table 4. Our results are similar to those previously reported by other studies involving ten healthy controls [36, 37]. For the genu of the CC, body of the CC, splenium of the CC, and the ILF, the mean differences in the patients’ acute-to-subacute FA changes were larger than the corresponding regional RDCs for the control dataset: 0.15 vs. 0.02, 0.08 vs. 0.06, 0.031 vs. 0.030, and 0.06 vs. 0.05, respectively. However, for the cingulum bundle, the patients’ longitudinal FA changes were within the control’s regional RDC: 0.07 vs. 0.09.

**Table 4:** Comparison of DTI measurements of FA in the control dataset (*n* = 1 subject, *n* = 9 DTI scans) and TBI dataset (*n* = 22 TBI patients, *n* = 11 acute scans, *n* = 11 subacute scans)

	Control DTI Scan #	Genu CC FA	Body CC FA	Splenium CC FA	ILF FA	CB FA
Session 1	1	0.81	0.65	0.82	0.55	0.65
Session 2	2	0.81	0.64	0.82	0.58	0.61
Session 3	3	0.80	0.62	0.82	0.55	0.58
Session 4	4	0.80	0.66	0.83	0.57	0.63
Session 5	5	0.81	0.67	0.82	0.56	0.65
	6	0.82	0.64	0.80	0.57	0.64
Session 6	7	0.80	0.66	0.80	0.58	0.69
	8	0.79	0.70	0.83	0.61	0.64
	9	0.80	0.65	0.82	0.57	0.61
Control Mean ± SD		0.80 +/− 0.01	0.65 +/− 0.02	0.82 +/− 0.01	0.57 +/− 0.02	0.63 +/− 0.03
Control CV		1.1 %	3.4 %	1.3 %	3.2 %	4.9 %
Control RDC		0.02	0.06	0.030	0.05	0.09
Patient Mean Difference Acute-to-Subacute FA [95 % CI]		−0.15 [−0.25, −0.04]	−0.08 [−0.20, 0.04]	−0.031 [−0.11, 0.05]	−0.06 [−0.10, 0.01]	−0.07 [−0.12, −0.02]

Abbreviations: *CB* cingulum bundle, *CC* corpus callosum, *CV* coefficient of variation, *FA* fractional anisotropy, *ILF* inferior longitudinal fasciculus, *RDC* reproducibility coefficient. Note that for the splenium of the CC, the RDC value and the patient mean difference between acute and subacute FA are shown with three decimal places to demonstrate that the latter value was larger in magnitude than the former

## Discussion

The main findings of this study are that white matter FA declined significantly between the acute and subacute stages of TBI, and subacute FA correlated more consistently with DRS than acute FA. While a longitudinal decline in FA has been previously observed between the subacute and chronic stages of TBI [11, 33, 38, 39] and the acute and chronic stages of TBI [27], we identified an early decline in FA between the acute and subacute stages. These longitudinal DTI observations may help to elucidate the complex pathophysiological effects of TAI on white matter microstructure, while also providing preliminary evidence that the optimal timing of DTI data acquisition for functional outcome prediction using FA may be in the subacute stage of TBI rather than at acute time points within 1 week of injury.

The pathophysiological basis for a decline in FA during the acute-to-subacute stages of TBI can be understood by considering how TAI affects the ratio of intracellular to extracellular water content. Histopathological studies in animals [9, 40–42] and humans [42–44] have demonstrated that acute TAI is a dynamic process with variable effects on axonal microstructure. If shear strain forces are severe, primary axotomy and mechanical disruption of the blood–brain barrier may occur acutely. Both of these processes may cause water to accumulate in the extracellular space, where water diffusion is less directional, leading to decreased FA. However, if shear strain forces are mild or moderate, TAI may produce incomplete axonal injury characterized by disruption of neurofilaments, loss of ionic homeostasis, and mitochondrial dysfunction, but not acute axotomy [42]. This scenario can result in either secondary axotomy or recovery of axonal structural integrity. In this incomplete form of TAI, water may accumulate within the intracellular compartment [42, 43, 45], leading to more directional water diffusion along the axis of the axon, and hence increased FA. It should be noted, however, that even when intracellular edema occurs in incomplete TAI, the impact on FA theoretically varies depending upon the degree of microtubule and neurofilament disruption within the axon. Alternatively, intramyelin edema may lead to more restricted radial diffusion, which could also lead to increased FA.

In contrast to these heterogeneous FA changes during the acute stage of TBI, which depend upon microstructural white matter alterations that vary with TAI severity, FA changes during the subacute stage appear to be more consistent. Regardless of whether axons undergo acute primary axotomy with subacute Wallerian degeneration, secondary axotomy with subacute Wallerian degeneration, or incomplete injury with subsequent recovery of axonal structural integrity, each of these longitudinal processes is expected to cause a decline in the ratio of intracellular to extracellular water content, and therefore a decline in FA. Indeed, we observed a decline in FA in all white matter ROIs during the transition from the acute to the subacute period, although these declines in FA only reached statistical significance for 3 of the 11 white matter ROIs. Furthermore, the longitudinal decline in FA only remained significant for 2 ROIs – the genu of the corpus callosum and the inferior longitudinal fasciculus – when we controlled for regional FA variability observed in the control DTI dataset. The small number of white matter regions with significant longitudinal FA changes may be explained either by a lack of statistical power related to our small sample size, or by a heightened vulnerability of these white matter regions to the biomechnical shearing forces of TAI. Notably, the proposed pathophysiological basis for a longitudinal decline in FA is supported by our ADC analyses. ADC values were lower during the acute period than the subacute period in all 11 white matter ROIs, with this acute-to-subacute increase in ADC reaching statistical significance for the uncinate fasciculus. Similarly, in two prior studies that found increased white matter FA during the acute stage of TBI, decreased acute ADC values were also observed [24, 25], suggesting acute restriction of water diffusion within the intracellular compartment.

The variability of FA changes in acute TAI also helps explain our observations regarding the relationship between acute FA and outcome. Just as prior studies have shown an unpredictable relationship between acute FA and TBI outcome [12, 13, 24, 25], we found that during the acute period, higher FA in the body and splenium of the CC correlated with good outcome, whereas higher FA in the genu of the CC correlated with poor outcome. These findings may be understood by considering the severity of TBI in the patients in each analysis. In two prior studies that demonstrated a correlation between an acute increase in FA and poor outcome [24, 25], the severity of TBI in all patients was mild, in which case incomplete axonal injury, and hence increased FA, are more likely. In contrast, in two studies that showed a correlation between an acute decline in FA and poor outcome, the severity of TBI was moderate-to-severe [12, 13], in which case primary axotomy and blood–brain barrier disruption, and hence decreased FA, are more likely. In our study, the inclusion of patients with complicated mild (*n* = 3), moderate (*n* = 1) and severe (*n* = 7) TBI may therefore explain why the correlations between acute FA and DRS were variable.

It is also important to consider that all of the ROIs demonstrating correlations between regional FA and outcome were located in the corpus callosum (genu, body, and splenium). We speculate that these correlations exist not only because callosal TAI lesions are common in patients with higher grades of “diffuse axonal injury” [2], or because the callosum is particularly susceptible to biomechanical shear-strain forces [46], but also because the callosum is a conduit for multiple white matter bundles that undergo TAI within the cerebral hemispheres. Indeed, patients with severe TBI (seven of 11 patients in this study) have consistently been shown to have diffuse bihemispheric TAI lesions – from the initial histopathological studies of TAI in the 1950s [47] to modern DTI studies [14, 48, 49]. Thus, corpus callosum FA may act as both a biomarker of focal TAI in the callosum as well as a surrogate marker for widespread white matter injury throughout the cerebral hemispheres. This interpretation is consistent with our observation that subacute FA correlates with outcomes more reliably than does acute FA, because the subacute period is when hemispheric white matter injury is more likely to affect callosal FA via Wallerian degeneration of transcallosal axons. Notably, only FA in the splenium of the corpus callosum remained significantly associated with DRS score after a second rater retraced the ROIs, and this was the ROI for which the interrater coefficient did not vary from the acute to subacute period (0.92 to 0.93). In contrast, in the genu and body of the CC, there was more variability between the interrater coefficients for the acute DTI scans and the subacute DTI scans (0.53 to 0.94 and 0.60 to 0.84, respectively). These findings further suggest that the timing of DTI data acquisition may influence the reproducibility of FA measurements in some neuroanatomic regions (e.g. genu and body of the CC) more than others (e.g. splenium of the CC).

Several limitations should be considered in interpreting the results from this study. First, our sample size was small (*n* = 11) and one patient was missing DRS data. Thus, our analyses may not have been adequately powered to detect statistically significant correlations between FA and DRS scores. Similarly, the exclusion of DTI data from some ROIs due to contusion or artifact from intracranial hardware may have limited the statistical power. Second, the spatial resolution (slice thickness 5–6 mm) and angular resolution (6–25 diffusion-encoding directions) were low due to the need for rapid data acquisition in a clinical setting. Third, DTI data were acquired on several scanners that were upgraded at our institution over the 4-year study period, potentially causing variability in the FA measurements. Although all 22 DTI scans were performed at the same field strength (1.5 Tesla) and with the same *b* value (1000 s/mm^2^), two of the 11 study subjects underwent acute and subacute scans with different numbers of diffusion-encoding directions (6 and 25 respectively), a difference that may alter FA measurements [34]. Nevertheless, the identification of significant longitudinal FA changes and correlations with outcome despite these potential confounders indicates that our findings may be generalizable to clinical settings in which DTI sequence parameters are not strictly controlled. Furthermore, we observed that longitudinal changes in corpus callosum FA and inferior longitudinal fasciculus FA in the patient cohort were larger than the regional FA RDC observed in the control DTI dataset, although longitudinal FA changes in the cingulum bundle were within the control’s range of regional FA RDC. A fourth limitation of this study is that while the outcome data were obtained prospectively, the DTI data were analyzed retrospectively, without standardized timing of the acute and subacute DTI scans. Finally, although our study population included patients with mild TBI according to admission GCS criteria (13–15), all of these patients met criteria for “complicated” mild TBI and thus our results cannot necessarily be generalized to patients with “uncomplicated” mild TBI.

## Conclusions

In summary, this exploratory longitudinal study provides preliminary evidence that the optimal timing of DTI data acquisition for TBI prognostication may be in the subacute stage of injury (i.e. after day 7). Our findings are consistent with prior studies that demonstrated a variable relationship between acute FA and outcome, in comparison to the more predictable relationship between subacute FA and outcome. Subacute FA measurements may be more likely to reflect the structural integrity of axons that have been affected by TAI and less likely to be confounded by intracellular or extracellular edema. Ultimately, decisions regarding the timing of DTI data acquisition need to balance the competing goals of delaying data acquisition until edema has resolved and acquiring DTI data early enough that they can be useful for guiding patient management. Given the retrospective nature of this study and the limited precision of our FA measurements due to variability in the DTI sequence, our findings should be considered hypothesis-generating for future studies. Prospective longitudinal studies of larger cohorts with a standardized DTI sequence are needed to further elucidate the dynamic pathophysiologic effects of TAI on white matter FA and the optimal timing of DTI for TBI prognostication.
